# Comparative genomics and transcriptomics in ants provide new insights into the evolution and function of odorant binding and chemosensory proteins

**DOI:** 10.1186/1471-2164-15-718

**Published:** 2014-08-26

**Authors:** Sean K McKenzie, Peter R Oxley, Daniel JC Kronauer

**Affiliations:** Laboratory of Insect Social Evolution, The Rockefeller University, 1230 York Avenue, 10065 New York, NY USA

**Keywords:** Chemical communication, Sociogenomics, Formicidae, Chemosensation, OBP, CSP, Comparative genomics

## Abstract

**Background:**

The complex societies of ants and other social insects rely on sophisticated chemical communication. Two families of small soluble proteins, the odorant binding and chemosensory proteins (OBPs and CSPs), are believed to be important in insect chemosensation. To better understand the role of these proteins in ant olfaction, we examined their evolution and expression across the ants using phylogenetics and sex- and tissue-specific RNA-seq.

**Results:**

We find that subsets of both OBPs and CSPs are expressed in the antennae, contradicting the previous hypothesis that CSPs have replaced OBPs in ant olfaction. Both protein families have several highly conserved clades with a single ortholog in all eusocial hymenopterans, as well as clades with more dynamic evolution and many taxon-specific radiations. The dynamically evolving OBPs and CSPs have been hypothesized to function in chemical communication. Intriguingly, we find that seven members of the conserved clades are expressed specifically in the antennae of the clonal raider ant *Cerapachys biroi*, whereas only one dynamically evolving CSP is antenna specific. The orthologs of the conserved, antenna-specific *C. biroi* genes are also expressed in antennae of the ants *Camponotus floridanus* and *Harpegnathos saltator*, indicating that antenna-specific expression of these OBPs and CSPs is conserved across ants. Most members of the dynamically evolving clades in both protein families are expressed primarily in non-chemosensory tissues and thus likely do not fulfill chemosensory functions.

**Conclusions:**

Our results identify candidate OBPs and CSPs that are likely involved in conserved aspects of ant olfaction, and suggest that OBPs and CSPs may not rapidly evolve to recognize species-specific signals.

**Electronic supplementary material:**

The online version of this article (doi:10.1186/1471-2164-15-718) contains supplementary material, which is available to authorized users.

## Background

Eusocial insects, and ants in particular, are becoming increasingly popular models for the genetics and neurobiology of social behavior [1, 2]. Chemical communication is the predominant mode of coordination in insect societies and as such is essential for insect eusociality [3]. Understanding the molecular and neural underpinnings of social insect chemosensation may therefore provide broad insight into social evolution and behavior [4]. In insects, odorants are detected by receptor proteins embedded in olfactory and gustatory receptor neurons, which are located in porous sensilla and surrounded by sensillar lymph. Odorants enter the sensilla through the pores and diffuse through the sensillar lymph to the receptor proteins, binding and activating these proteins to produce action potentials in the receptor neurons. A variety of accessory proteins in the receptor lymph are also involved in this process, including two families of olfaction-related small soluble proteins (ORSSPs) – the odorant binding proteins (OBPs) and the chemosensory proteins (CSPs). These proteins help solubilize hydrophobic chemicals and may aid in odor detection, discrimination and coding [5, 6].

Although several studies have demonstrated the importance of ORSSPs in insect chemosensation (e.g. [7–10]), their exact function has been hard to pinpoint. Additionally, many proteins in both families are expressed in non-olfactory tissues and have been linked to such diverse functions as developmental patterning, internal pheromone transport and release, and leg regeneration [11–13]. Both OBPs and CSPs have evolved dynamically between insect orders, with high gene birth and death rates and highly variable copy numbers in different genomes [14]. On the other hand, genes in both protein families appear to be more conserved at lower taxonomic levels, with mostly single-copy orthology in the genus *Drosophila* and family Aphidae, and a mix of single-copy orthology and dynamic evolution in the family Culicidae and in the wasp infraorder Aculeata (which contains all social hymenopterans including ants) [15–19]. No study has yet examined the links between evolutionary history and tissue localization, nor systematically examined ORSSP expression patterns in a broadly comparative context.

We therefore decided to investigate the evolution of ORSSP expression patterns in ants using genomics, phylogenetics, and transcriptomics. Methods include manual re-annotation of ant OBP and CSP gene families, manual annotation of OBP and CSP genes in the transcriptome of the paper wasp *Polistes canadensis,* extensive phylogenetic analyses, and a 14-library tissue-specific RNA-seq data set from the clonal raider ant *Cerapachys biroi*, supplemented by a re-analysis of four libraries of previously published RNA-seq data from the ants *Harpegnathos saltator* and *Camponotus floridanus* [4]. We find that a stable set of eight conserved genes (three CSPs and five OBPs) present in single copy orthology across Aculeata are moderately to highly expressed in the antennae of the three divergent ant species, along with four to five genes from more dynamically evolving lineages previously hypothesized to act in chemical communication. Interestingly, seven of the conserved genes are expressed specifically in the antennae of *Cerapachys biroi*, while four of five dynamically evolving genes are more broadly expressed. These results indicate that antennal ORSSPs fulfill important and conserved roles in olfaction, rather than evolving rapidly to recognize specific ligands.

## Results

### Phylogenetic analysis

Within the odorant binding and chemosensory protein families, sequence divergence is high, with average amino acid identities of only 17% for insect OBPs and 34% for arthropod CSPs [14]. Because of the analytical challenges associated with highly divergent sequences, we inferred phylogenies for OBPs and CSPs within the eusocial Hymenoptera using Bayesian co-estimation of sequence alignment and phylogeny, as well as traditional multiple sequence alignment with maximum likelihood tree inference. Table 1 lists all eusocial hymenopteran species examined and the number of OBP and CSP gene models in each species. Additionally, we constructed maximum likelihood phylogenies for OBPs and CSPs across Arthropoda.

**Table 1:** **Number of annotated OBPs and CSPs in different species of eusocial Hymenoptera**

	OBPs	CSPs
*C. biroi*	15 (2)	15
*H. saltator*	13 (13)	12 (1)
*C. floridanus*	13 (13)	13 (1)
*P. barbatus*	16 (1)	11
*L. humile*	13 (1)	15
*S. invicta*	17 (1)	21
*P. canadensis*	9 (9)	9 (9)
*A. mellifera*	21	6

Numbers in parentheses represent numbers of genes that were either newly annotated or re-annotated as part of the current study.

**Figure 1:**
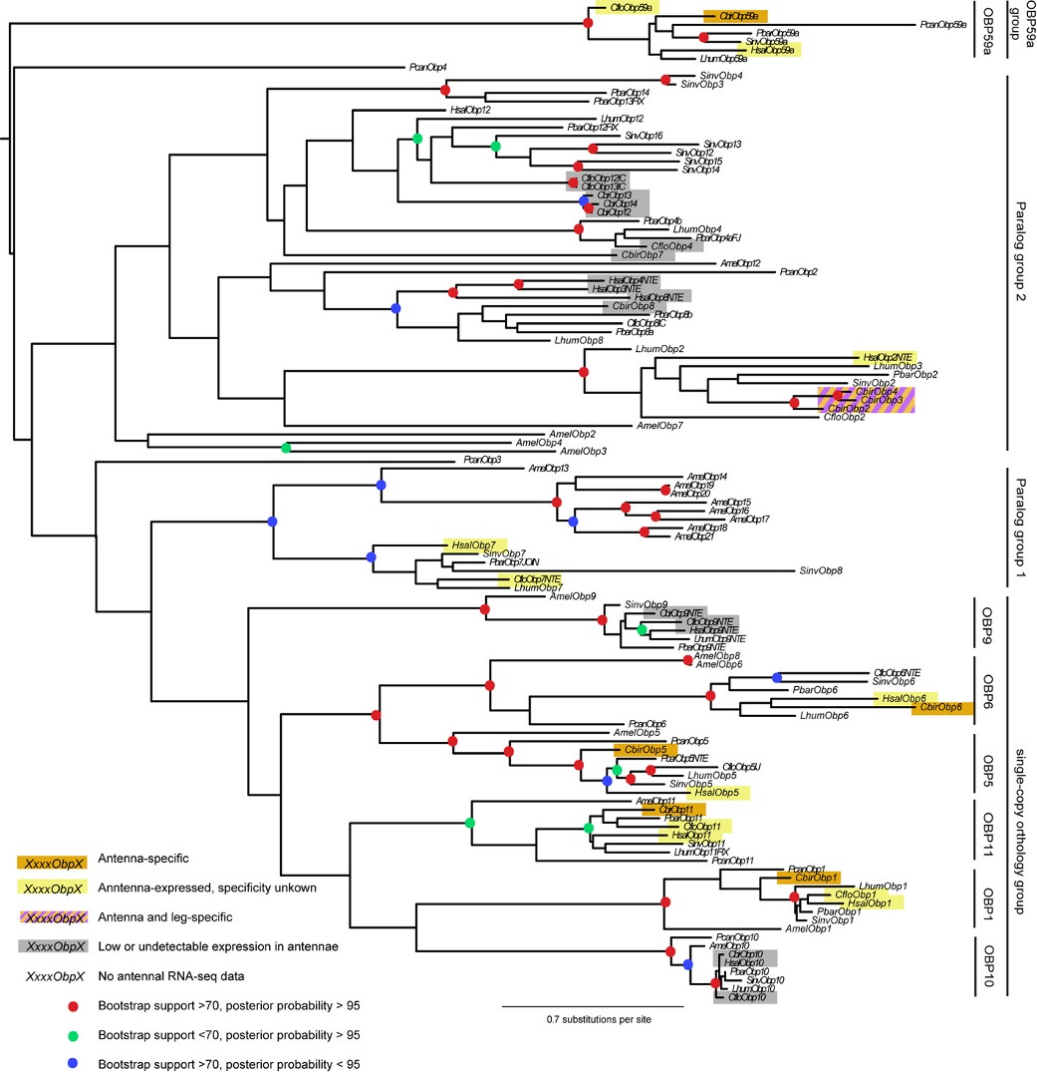
**Maximum likelihood phylogeny of odorant binding proteins in social Hymenoptera.** Phylogenetic hypothesis constructed using RAxML [73]. Bootstrap support was calculated with 100 RAxML rapid bootstrap replicates, and posterior probabilities were calculated using BAli-Phy [67]. Pcan: *Polistes canadensis*, Amel: *Apis mellifera*, Hsal: *Harpegnathos saltator*, Cbir: *Cerapachys biroi*, Lhum: *Linepithema humile*, Cflo: *Camponotus floridanus*, Pbar: *Pogonomyrmex barbatus*, Sinv: *Solenopsis invicta*.

Topologies were largely congruent across our analyses, although support values for individual nodes varied considerably and were generally low. Our analyses of eusocial hymenopteran OBPs consistently recovered four major groups: one containing well-supported clades of strict single-copy orthologs (except *AmelObp6*/*AmelObp8*, which are products of a recent duplication) (we will refer to this group as the “single-copy orthology group”), one containing the *Apis mellifera* ‘c-minus’ expansion with a few ant paralogs (“paralog group 1”), one containing mostly ant species-specific expansions with a few *A. mellifera* and *P. canadensis* paralogs (“paralog group 2”), and one containing a single clade of highly conserved OBPs, highly divergent from other OBPs and present in single-copy orthology in all species except *A. mellifera* (“*Obp59a* group”) (Figure 1). The *Obp59a* group is so divergent from other aculeate OBPs that it had been missed entirely in all previous ant OBP annotations. The group is orthologous to *Drosophila melanogaster Obp59a*, and we therefore decided to name it accordingly. Two *P. canadensis* OBPs (*PcanObp3* and *PcanObp4*) did not fit into any of these groups and likely represent ancestral groups lost in the ants and the honey bee.

Combined analysis of our eusocial hymenopteran dataset and other insect species revealed that the paralog groups are young, Aculeata-specific expansions. On the other hand, all ortholog clades within the ortholog group and the *Obp59a* group have *Nasonia* orthologs, and many also have non-Hymenoptera orthologs (Additional file 1: Figure S1). This indicates that the single-copy ortholog groups date back at least as far as the split between Hymenoptera and the rest of Endopterygota, ca. 350 MYA [20], while the other two groups are most likely younger than the most recent common ancestor of *Nasonia* and Aculeata, ca. 250 MYA [21].

**Figure 2:**
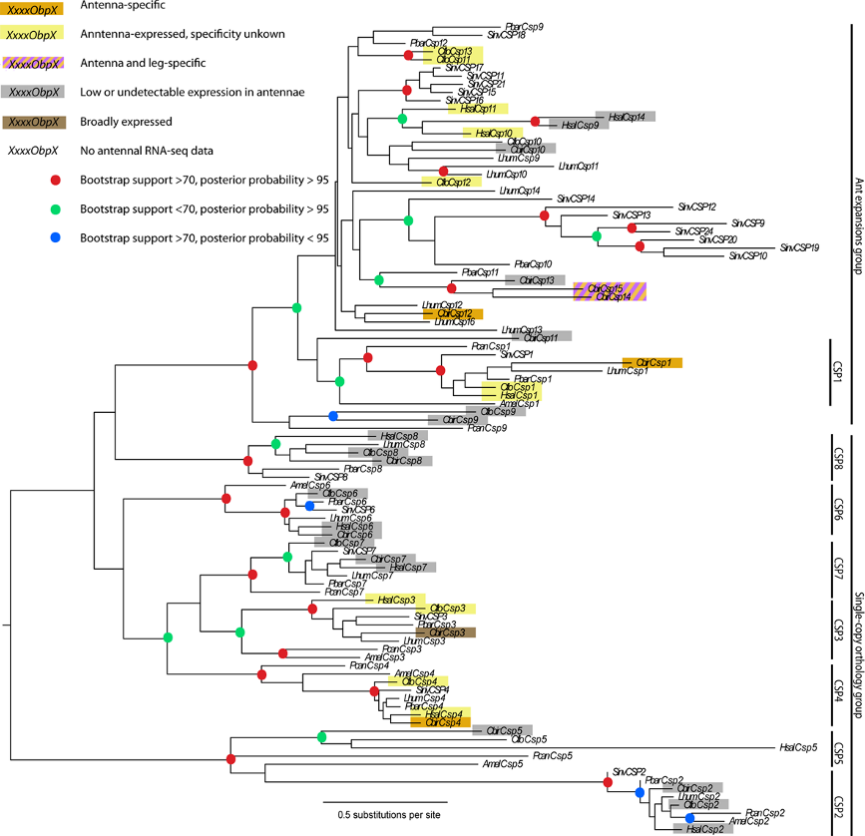
**Maximum likelihood phylogeny of chemosensory proteins in social Hymenoptera.** Phylogenetic hypothesis constructed using RAxML [73]. Bootstrap support was calculated with 100 RAxML rapid bootstrap replicates, and posterior probabilities were calculated using BAli-Phy [67]. Pcan: *Polistes canadensis*, Amel: *Apis mellifera*, Hsal: *Harpegnathos saltator*, Cbir: *Cerapachys biroi*, Lhum: *Linepithema humile*, Cflo: *Camponotus floridanus*, Pbar: *Pogonomyrmex barbatus*, Sinv: *Solenopsis invicta*.

Analyses of eusocial hymenopteran CSPs showed evolutionary patterns consistent with Kulmuni et al. [18] and similar to OBPs. Two well-supported groups were recovered, one containing exclusively well-supported clades of single-copy orthologs with *A. mellifera* and/or *P. canadensis* orthologs, and one containing mostly ant species-specific expansions, but encompassing a single clade of single-copy orthologs including an *A. mellifera* ortholog (Figure 2). Among the single-copy orthologs was a novel clade missed in previous ant CSP annotations, possibly orthologous to *AmelCsp5*. A full-length potential ortholog of *Csp5* was present in the ants *Harpegnathos saltator*, *Cerapachys biroi*, and *Camponotus floridanus*, but present only as a highly pseudogenized fragment in *Linepithema humile* and missing in all myrmicine ants. Comparison with other arthropod CSPs showed that the paralogous ant-specific expansions group is a Hymenoptera-specific radiation, while the single-copy ortholog clades are much older and diverged before hexapods split from the rest of Pancrustacea, ca. 480 MYA [22] (Additional file 2: Figure S2).

### Evolutionary dynamics

To quantify the dynamism of the ant expansion and paralog groups compared to the single-copy orthology and *Obp59a* groups, we conducted statistical gene birth and death analyses for all four OBP groups (single-copy orthology groups, paralog group 1, paralog group 2, and *Obp59a* group) and both CSP groups (ant expansion group and single copy orthology group) using the CAFE and JPrIME-DLRS analyses [23, 24]. CAFE uses maximum likelihood modeling and ancestral state reconstruction of gene copy numbers and is thus likely conservative as it doesn’t take the gene tree (and thus parallel paralogy) into account. JPrIME-DLRS estimates birth and death rates jointly with a gene tree given a species tree, using the DLRS (duplication, loss, rates, and sequence evolution) model within a Bayesian MCMC framework.

As expected, CAFE estimated lower birth and death rates than JPrIME-DLRS (Table 2). Both methods showed dynamic evolution in the paralog and ant expansion groups, with birth and death rates ranging from 0.0028 to 0.0071 as estimated by CAFE and 0.0052 to 0.013 as estimated by JPrIME-DLRS. Estimates of birth and death rates were often more than an order of magnitude lower for the single-copy orthology and *OBP59a* groups, with estimates of 4.1x10^-11^-0.001 and 0.0008-0.002 for CAFE and JPrIME-DLRS, respectively.

**Table 2:** **Gene birth and death rates for each group of OBPs and CSPs as estimated by CAFE and JPrIME-DLRS**

	CAFE λ	CAFE μ	DLRS λ	DLRS μ
PG2 OBPs	0.0028	0.0034	0.0067 (0.0034-0.012)	0.007 (0.0022-0.017)
PG1 OBPs	0.0071	0.003	0.0063 (0.0023-0.012)	0.0052 (0.00092-0.013)
SCG OBPs	0.00039	4.1x10^-11^	0.0013 (0.00054-0.0023)	0.0016 (0.00068-0.0029)
59a OBPs	9.6x10^-10^	0.001	0.0008 (3x10^-9^-0.0047)	0.002 (3x10^-5^-0.0088)
AEG CSPs	0.005	0.0044	0.0095 (0.0059-0.014)	0.013 (0.008-0.19)
SCG CSPs	3.7x10^-9^	0.00094	0.001 (0.00037-0.0019)	0.0013 (0.00039-0.0026)

λ = gene birth rates, μ = gene death rates. Numbers in parentheses are 95% highest posterior density intervals for the JPrIME-DLRS run. PG1: paralog group 1, PG2: paralog group 2, 59a: *Obp59a* group, SCG: single-copy orthology group, AEG: ant expansions group.

Although several studies have examined selective pressures in both OBPs and CSPs [17, 18, 25], these studies have used different methods and different taxonomic sampling, making direct comparisons impossible. We thus conducted a suite of selection tests on both our OBP and CSP datasets using the codeml software in the paml package [26] (results summarized in Table 3). Site models of positive selection showed no significant positive selection family-wide for either CSPs or OBPs (M1a-M2a likelihood ratio test (LRT) and M7-M8 LRT, *P* > 0.05 for both tests). Clade tests for divergent selection showed that there was significant variance in selective pressures between the groups (clade model C vs. M2a_rel LRT, *P* < 0.001). Clade model C showed 79% and 62% of sites are under divergent selection in OBPs and CSPs, respectively. OBP paralog groups 1 and 2 and the CSP ant expansion group showed much higher dn/ds in the divergently selected site class than the OBP single-copy orthology, *Obp59a*, and CSP single-copy orthology groups (dn/ds of 0.5, 0.47, 0.5, 0.19, 0.2, and 0.24, respectively). No group showed positive selection (dn/ds > 1) in the divergently selected site class, indicating that relaxed selection was the predominant selective force in all groups.

**Table 3:** **Negative log likelihoods for different CodeML models and** ***P*** **values of LRT tests comparing various models**

	-Ln(likelihood)									***P***				
	NsitesM0	NsitesM1	NsitesM2	NsitesM7	NsitesM8	CmC	M2a_Rel	MAalt	MAnull	M1a-vs-M2a	M7-vsM8	CmC-vs-M2a_rel	MAalt-vs-M1a	MAalt-vs-MAnull
All OBPs	43061.9	42980.1	42980.1	42629.2	42628.0	42600.1	42724.8			1	0.201	< 0.001		
All CSPs	26669.8	26491.3	26491.2	26174.2	26171.9	26200.1	26257.7			1	0.086	< 0.001		
PG2 OBPs	17839.3	17663.0	17658.7	17579.6	17576.2			42772.8	42772.8	0.02	0.04		< 0.001	1
PG1 OBPs	4397.4	4355.7	4355.7	4353.8	4351.6			42918.3	42918.3	1	0.09		< 0.001	1
59a OBPs	4406.6	4357.1	4357.1	4337.3	4337.3			42975.9	42975.9	1	1		0.059	1
SCG OPBs	15337.6	15319.7	15319.7	15147.7	15147.7			42955.1	42955.1	1	1		< 0.001	1
AEG CSPs	13000.5	12759.8	12754.0	12651.3	12645.5			26295.9	26295.9	0.009	0.009		< 0.001	1
SCG CSPs	13710.2	13597.0	13597.0	13436.5	13434.4			26437.5	26437.5	1	0.1		< 0.001	1

PG1: paralog group 1, PG2: paralog group 2, 59a: *Obp59a* group, SCG: single-copy orthology group, AEG: ant expansions group.

We used branch-site tests of positive selection as described by Zhang *et al.* [27] to test for positive selection affecting each group. Zhang *et al.*’s branch-site test 1 (model A vs. M1a) can be positive when positive selection or relaxed selection is occurring along specified branches, while branch-site test 2 (model A alternative vs. model A null; also known as the branch-site test for positive selection) tests specifically for positive selection along specified branches. We ran branch-site tests for each group by setting all branches within each group in turn as foreground branches. Consistent with our clade model results, branch-site test 1 was significant for all groups except *Obp59a* (LRT, *P* < 0.001 for all groups except *Obp59a*, *P* = 0.059), but all groups were non-significant for test 2 (LRT, *P* > 0.05) indicating relaxed selection operating on all groups but the *Obp59a* group. We also split our data and ran site tests for positive selection for each group analyzed separately. Interestingly, although branch-site tests for positive selection (test 2) were negative for all groups, site tests for positive selection for each group analyzed separately were significant for the ant expansion CSP group and OBP paralog group 2 (M1a-M2a and M7-M8 LRTs, *P* < 0.05 for all comparisons). This could indicate that divergent relaxed selection is swamping the signal of divergent positive selection in the branch-site tests for these two groups. Bayes Empirical Bayes (BEB) analysis identified two sites with dn/ds significantly greater than one in the ant expansion CSP group for the M8 model, one of which was also significant in the M2a model. No OBP paralog group 2 sites had dn/ds significantly greater than one in either M8 or M2a models according to the BEB analysis.

### Sex-specific antennal expression

**Figure 3:**
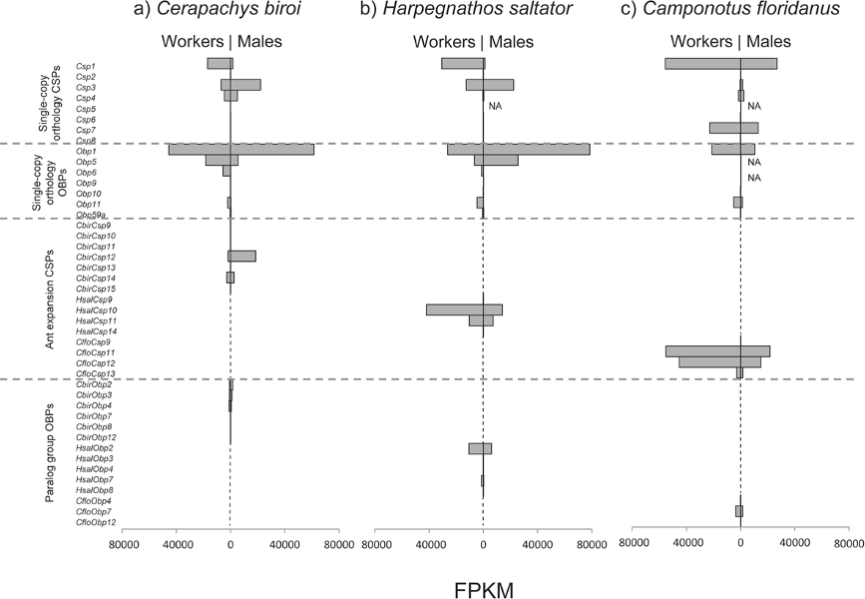
**Expression patterns of odorant binding proteins and chemosensory proteins in the antennae of males and workers of (a)** ***Cerapachys biroi*** **(b),** ***Harpegnathos saltator*** **, and (c)** ***Camponotus floridanus*** **.** NA indicates that the orthologous protein was not annotated in the official gene set for that species, and expression could therefore not be quantified.

Initially, we sequenced one cDNA library each for *Cerapachys biroi* male and female antennae to generate 80 million and 83 million 100 bp paired-end reads, respectively. Alignment and quantification of reads revealed high levels of transcription for subsets of both OBPs and CSPs (Figure 3a). Among the genes in the paralog clades, *CbirCsp12* was the only one expressed at high levels in antennae. Conversely, many genes in the ortholog clades were expressed at high levels in antennae; *CbirCsp3* and *CbirObp1* were non-significantly enriched in male vs. female antennae, while *CbirCsp1, CbirObp5, CbirObp6*, and *CbirObp11* were expressed at higher levels in the female antennae, and this was significant for *CbirObp11* (FDR adjusted *P*-value = 0.02; all others *P* > 0.05). *CbirCsp4*, *CbirCsp14*, *CbirObp2*, *CbirObp3*, and *CbirObp4* were found at moderate to low levels in both male and female antennae (Figure 3a).

A reanalysis of previously published sex-specific antennal RNA-seq data from the ants *Harpegnathos saltator* and *Camponotus floridanus* [4] revealed expression profiles highly similar to those of *C. biroi* (Figure 3b and c). This was especially true for *H. saltator*, which showed nearly identical expression patterns for single-copy orthologs, except for a reversal in sex-specific enrichment for *Obp5* and very low expression of *Csp4*. Two members of single-copy orthology clades, *Obp5* and *Obp6*, are not annotated in the *C. floridanus* official gene set, as the assembly of the respective genomic regions is fragmentary. Thus, their expression levels were not included in the dataset. However, all other orthologs of proteins expressed in *C. biroi* antennae are likewise expressed in *C. floridanus* antennae. Several other genes are expressed at high levels in *C. floridanus* antennae, notably *CfloCsp7*, *CfloObp7,* and several CSPs in the paralog clade. Intriguingly, no annotated OBP or CSP was noticeably male-enriched in *C. floridanus* antennae in our analysis.

### Female tissue-specific expression

**Figure 4:**
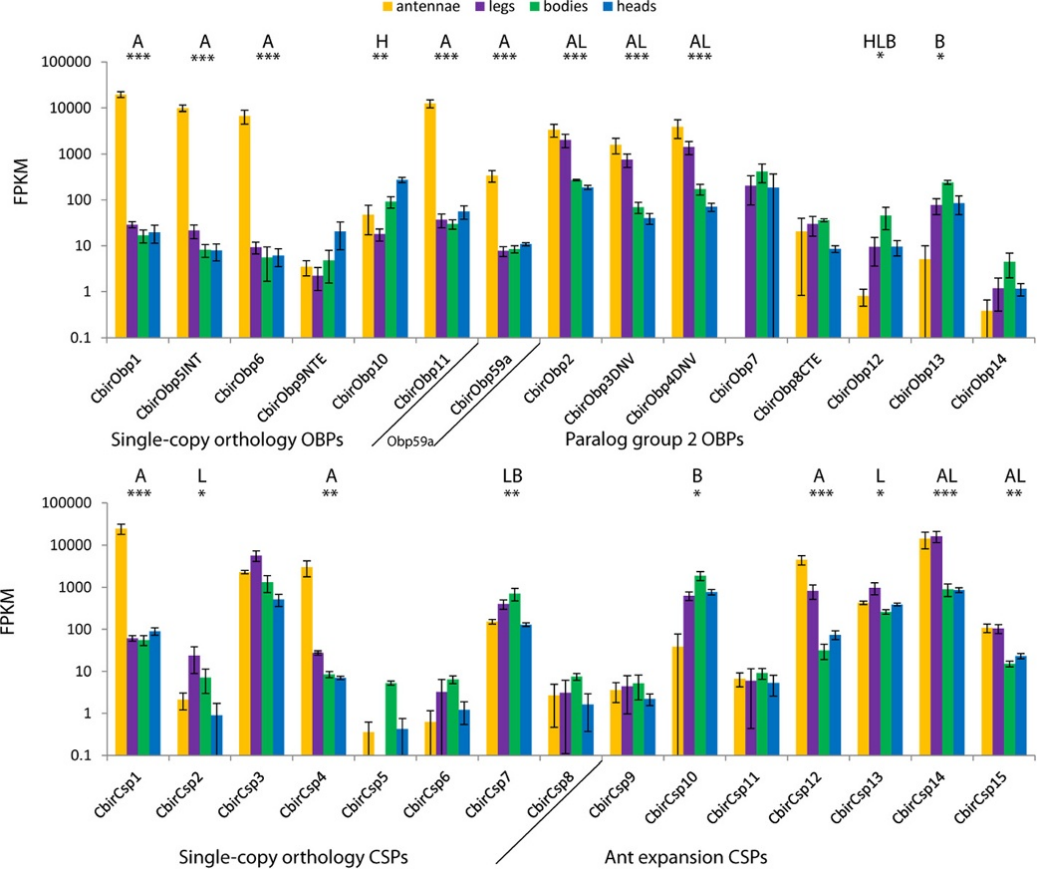
**Expression levels of OBPs and CSPs in the antennae, legs, bodies (thorax + abdomen), and heads (minus antennae) of** ***Cerapachys biroi*** **workers.** Data are shown log-transformed, error bars indicate standard deviations. Letters indicate tissues significantly enriched compared to all remaining tissues: A = antennae, L = legs, B = bodies, H = heads. Asterisks indicate maximum FDR adjusted *P* value of enriched vs. other tissues: * < 0.05, ** < 0.01, *** < 0.001. Expression was calculated with the Cufflinks software, and differences in expression were tested for significance using CuffDiff (Trapnell et al. 2013). Three biological replicates were analyzed for each tissue type.

In order to test which *C. biroi* OBPs and CSPs are specifically expressed in antennae, we sequenced three biological replicates of cDNA libraries from female antennae, heads (minus antennae), legs, and bodies (abdomen + thorax without legs). We obtained an average of 18 million 100 bp single-end reads per library. Additional sequencing of male samples was not possible, because males are produced exceedingly rarely in *C. biroi* [28]. *CbirObp5*, *CbirObp6*, *CbirObp11* and *CbirObp59a*, as well as *CbirCsp1*, *CbirCsp4*, and *CbirCsp12* were significantly enriched in the antennae compared with other tissues (*P* < 0.006 for all comparisons involving antennae; Figure 4). All female antenna-specific genes except *CbirCsp12* and *CbirObp59a* belong to conserved single-copy ortholog clades with *A. mellifera* and *P. canadensis* orthologs (Figures 1 and 2) and with moderate to high expression in *H. saltator* and/or *C. floridanus* antennae (Figure 3). *CbirObp59a* does belong to a conserved single-copy ortholog clade but has no *A. mellifera* ortholog (Figure 1) and is expressed only at low levels in the antennae of all three ant species (Figure 3). Expression of *CbirCsp3*, also a single-copy ortholog, was not enriched in worker antennae (Figure 4), but given its male-biased expression (Figure 3) it might be antenna-enriched in males. *CbirObp2*, *CbirObp3*, *CbirObp4*, *CbirCsp14,* and *CbirCsp15* were most significantly enriched in antennae and legs vs. heads and bodies (*P* > 0.05 for all antennae vs. legs comparisons, *P* < 0.0005 for all antennae/legs vs. head/body comparisons). *CbirCsp10*, *CbirCsp7*, and *CbirObp13* were most highly expressed in bodies (*P* < 0.05 for all body vs. antennae/legs/head comparisons). *CbirObp10* was expressed nearly exclusively in heads (*P* < 0.05 for all head vs. antennae/legs/body comparisons). *CbirObp7* was expressed at low levels in heads, bodies and legs, but expression was only significantly different between bodies and antennae (*P* = 0.015). The rest of the genes had only a few reads that mapped to them (<50 FPKM), indicating that these genes are either expressed only at low levels, or show high levels of expression only in other developmental stages or non-antennal tissues in males. Table 4 shows how many OBPs and CSPs are expressed at greater than 50 FPKM in each tissue.

**Table 4:** **Number of OBPs and CSPs expressed in each tissue at > 50 FPKM in females**

	antennae	head	bodies	legs
OBPs	8 (5)	6 (1)	6 (1)	5 (0)
CSPs	8 (3)	7 (0)	6 (1)	8 (2*)

Numbers in parentheses are the numbers of genes specifically expressed in that tissue (i.e. significantly higher in that tissue than in all other tissues; see Figure 4).

*One CSP (*CbirCsp2*) is specifically expressed in legs and therefore contributes to the count in parentheses, but is expressed at less than 50 FPKM in legs and so does not contribute to the count of genes expressed in legs.

## Discussion

Aculeate odorant binding and chemosensory proteins display similar phylogenetic patterns, with several highly conserved clades with single-copy orthologs in all or most species and one (CSPs) and two (OBPs) dynamically evolving clades with many species-specific expansions. In contrast to previous hypotheses [17, 18], it is primarily a conserved subset of single-copy orthologs which are expressed in ant antennae. In *Cerapachys biroi*, only one dynamically evolving CSP is expressed specifically in antennae, in contrast to five conserved OBPs and two conserved CSPs showing antenna-specific expression. This is also the first study to demonstrate antenna-specific expression of OBPs in ants, contradicting the hypothesis that CSPs alone are expressed antenna-specifically in ants [29]. Our results suggest that OBPs and CSPs primarily fulfill important conserved roles in ant olfaction rather than dynamically evolving to recognize species-specific odorants.

### Ants use both OBPs and CSPs for olfaction

Krieger and Ross [30] identified the first ant odorant binding protein in the fire ant *Solenopsis invicta*, *gp-9*/*SinvObp3*. However, this protein is not antenna specific, but rather broadly expressed in the hemolymph [31]. Ishida et al. [32] identified a single antenna-specific CSP in the Argentine ant *Linepithema humile*, and later Leal and Ishida [31] identified an antenna-specific CSP in *S. invicta*. Ozaki et al. [33] found a CSP in the antennae of *Camponotus japonicus*, which was shown to function in chemical nestmate recognition. All of these studies were limited to identifying a single major antennal protein, although studies in other organisms indicate that many binding proteins likely contribute to olfaction [25, 34]. Despite the scant evidence, Calvello et al. [29] proposed that ants may preferentially use CSPs rather than OBPs for olfaction. More recently, Gonzalez et al. [35] found an additional OBP (*SinvObp1*) in *S. invicta* antennae using shotgun proteomics. However, it was not tested whether this protein was antenna specific or expressed throughout the body, as is the case for *SinvObp3*.

Our systematic investigation of ORSSP expression revealed that OBPs as well as CSPs can be specifically expressed in the antennae of ants. Only a small subset of each protein family is antenna specific in workers, however, with only eight antenna-specific small soluble proteins in *C. biroi*. Five additional proteins expressed in *C. biroi* antennae and legs may be involved in gustatory chemosensation (see below). In contrast, there are between nine and sixteen OBPs, and between one and three CSPs thought to be involved in olfaction in the honey bee *Apis mellifera* [25, 36], and this has been considered a remarkably low number relative to dipterans, which possess 45 to 80 OBPs alone, most of which are expressed specifically in chemosensory organs [14, 34]. The low number of antenna-specific OBPs and CSPs in ants is an enigma, as ants show remarkable olfactory abilities and advanced chemical communication. As has been proposed for *A. mellifera*, ants may compensate for a lack of olfactory small soluble proteins by the expansion of chemosensory receptor genes. Indeed, ants possess more than twice as many odorant receptors (ORs) as *A. mellifera*, and well over four times as many as *D. melanogaster* [4, 37–41]. However, OR and ORSSP repertoires are not necessarily inversely correlated. For example, the red flour beetle (*Tribolium castaneum*) has more ORs than *A. mellifera*, and nearly three times the combined number of OBPs and CSPs [14, 42].

Three OBPs and two CSPs are expressed at moderate to high levels in both antennae and legs of *C. biroi* and may also be involved in gustatory chemosensation, especially as legs are known to be involved in taste in other species including the honey bee [43–45]. Alternatively, these genes may be generally associated with the cuticle but not serve chemosensory functions. For example, several *D. melanogaster* OBPs are highly expressed in the cuticle, but not specifically in the chemosensory hairs [34, 46]. Forêt et al. [36] hypothesized that some CSPs may function in cuticle synthesis. Antennae and legs are the most cuticle-rich tissues sequenced in our study, thus expression of these genes is directly correlated with relative cuticle abundance.

### Conservation of antennal expression in OBPs and CSPs

Both Gotzek et al. [17] and Kulmuni et al. [18] speculated that the more dynamically evolving OBPs and CSPs were more likely to be expressed in the antennae and involved in olfactory processes, especially pheromone perception. This is because pheromones themselves evolve rapidly and dynamically [47], and if any ORSSPs specifically recognized pheromones they should co-evolve with the respective pheromones. Our results contradict this hypothesis, showing that primarily a stable set of conserved OBPs and CSPs are expressed specifically in the antennae of ants. This is corroborated by the fact that two of three previously identified antennal-specific ORSSPs (*LhumCsp1* [32], *CjapCsp1* [33]) are in single-copy ortholog clades with the antenna-specific *CbirCsp1*. Although most relationships between OBP single-copy ortholog clades are poorly supported, the antennally expressed OBPs *Obp5* and *Obp6* form a well-supported clade in all analyses. Phylogenetic and expression data are thus consistent with transitions between antennal and non-antennal expression of OBPs occurring four times prior to the evolution of ants, with subsequent complete conservation of antennal expression among ants. CSPs *Csp1* and *Csp4* are not particularly closely related and likely represent independent transitions between antennal and non-antennal expression, and one to a few additional proteins in the rapidly evolving ant expansions CSP clade appear to be recruited to antennal expression.

The conservation of expression may extend even further than ants. One antenna-specific *C. biroi* CSP (*CbirCsp4*) and all but one antenna-specific *C. biroi* OBP (*CbirObp59a*) have antenna-specific orthologs in *A. mellifera* [25, 36]. *CbirCsp1* also has antenna-specific orthologs in *Polistes dominula* and *Vespa crabro* [29, 48]. An interesting case is *Obp59a*, which is present in all ants and is antenna specific in *C. biroi*, but missing in *A. mellifera* and thus previously missed in the ant OBP annotations. This protein is exceptional in two ways: it is the lowest expressed antenna-specific protein in *C. biroi*, and it is present in single-copy orthology in every single insect species examined by us and Vieira and Rozas [14] except *A. mellifera*. In *D. melanogaster*, *Obp59a* is highly enriched in heads (including antennae and palps) compared to all other tissues, and the respective protein has been detected in the antennae by proteomic studies [49, 50]. This protein may thus represent an extreme example of olfactory function conservation. Although Vieira and Rozas [14] highlight the parallels of this gene with ORCO, a universally conserved and highly expressed odorant co-receptor necessary for the function of all ORs, the lack of *Obp59a* in *A. mellifera*, combined with its low expression level in *C. biroi* antennae, does not support such a critical role.

It should also be noted that the ant orthologs of the *A. mellifera* c-minus clade (e.g. *HsalObp7* and *CfloObp7*, no phylogenetic relation to *CbirObp7*), which are moderately expressed in the antennae of *H. saltator* and *C. floridanus*, may also be specifically expressed in the antennae of ant species with functional copies of these genes. Only a small pseudogene fragment of the *C. biroi* ortholog remains (*CbirObpfrag4*), and, due to the lack of functional members of this clade in *C. biroi*, we could not determine antenna specificity.

### Functional implications

There is growing debate over whether OBPs and CSPs directly recognize odorants and contribute to odor coding or act as more or less specific odorant solubilizing agents [5, 6, 51, 52]. Selective binding of odorants by certain OBPs and CSPs initially indicated that they may indeed be essential for odorant recognition [53, 54]. Structural studies showing large conformational shifts upon odorant binding suggested a model wherein binding proteins specifically recognize odorants, alter conformation upon binding, and then directly activate ORs [55, 56]. This was seemingly confirmed by a study of the *D. melanogaster* OBP *Lush*, which showed that a recombinant LUSH protein stuck in bound conformation could directly activate a pheromone-sensitive odorant receptor [57]. However, other studies have called into question the proposed selectivity of OBP and CSP binding, as well as the implications of binding affinities for *in vivo* function [58, 59]. Additionally, a recent study has demonstrated that conformationally activated LUSH is not an *in vivo* ligand for pheromone-sensitive ORs, and suggested a broader role for LUSH in solubilizing multiple fly pheromones [52].

Given the startling array of chemicals different ant species use for communication [60], it seems unlikely that the few OBPs and CSPs expressed in chemosensory organs could selectively recognize a large proportion of pheromone components. Furthermore, pheromones are known to evolve rapidly and dynamically [47], and receptor proteins known to specifically recognize odorants also evolve rapidly and dynamically, with many gene births and deaths even within families and genera [4, 61]. The highly conserved nature of most antenna-specific OBPs and CSPs indicates that they are not evolving to specifically recognize new pheromones and likely do not specifically recognize other odorants. Although one to three dynamically evolving CSPs may be recruited for chemosensation in a given ant species, this number is clearly not sufficient to recognize all of the species-specific chemical signals. Our data suggest that, rather than specifically recognizing pheromones, most ant ORSSPs fulfill important, highly conserved, and more general roles in olfaction. [51]

## Conclusions

Contrary to previous expectations, both odorant binding proteins and chemosensory proteins are expressed specifically in antennae of the clonal raider ant *Cerapachys biroi* and therefore likely serve olfactory functions. Our findings show that an evolutionarily stable core group of highly conserved small soluble proteins are antennally expressed in ants. Additionally, we propose that a few proteins are also recruited from more dynamically evolving lineage-specific expansions, preferentially from the ant expansion CSP group. In total, between eight and fourteen OBPs and CSPs are potentially involved in chemosensation in the species we examined. Given the startlingly large array of pheromones used in ants, we suggest that there are too few small soluble proteins to specifically recognize individual pheromones. The striking evolutionary stability of antennal expression rather indicates that chemosensory ORSSPs participate in important and highly conserved processes. Future functional studies should address questions such as whether ligand binding and solubilization properties are conserved in the conserved antenna-specific small soluble proteins, and whether the few CSPs recruited from the more dynamically evolving ant expansion clade are involved in more dynamically evolving processes such as chemical communication.

## Methods

### Ants

Experimental colonies were maintained in airtight 12 x 12 x 12 cm plastic containers with a 2 cm deep plaster of Paris floor. Two connected circular recesses (25 mm in diameter, 2 mm deep) that were covered by a glass slide served as the nest chamber. Colonies were fed a diet of frozen fire ant (*Solenopsis invicta*) brood. Colonies of *C. biroi* alternate between brood care and reproductive phases in stereotypical cycles [62]. Because gene expression can vary significantly between these behavioral states [41], we attempted to minimize gene expression noise by standardizing age and behavioral stage for all female samples. For the first sequencing experiment (male and female antennal transcription), 25 one-month old females were collected from a colony (MLL4 in Kronauer et al. [28]) halfway into the brood care phase, while males were collected opportunistically from large stock colonies over four years (9 individuals from MLL1 and 2 individuals from MLL6). For the second experiment (tissue-specific sequencing), one-month old females were collected from colonies two days into the brood care phase. We sequenced four tissue-specific libraries (antennae, heads without antennae, legs, body (thorax and abdomen)) for each of three clonal lineages (MML1, MLL4, and MLL6 in Kronauer et al. [28]).

### Tissue dissection, RNA preparation, and sequencing

For the first experiment, live ants were flash frozen following collection and stored at -80°C. Antennae were dissected on dry ice and immediately transferred to 1.5 ml tubes on dry ice. Antennae were homogenized with a Qiagen TissueLyser II in Qiagen buffer RLT, and RNA was extracted using the RNeasy protocol (Qiagen). cDNA libraries were constructed using Illumina TrueSeq kits and samples were run on an Illumina HiSeq 2000 platform for 100 cycles with paired-end reads. Library preparation and sequencing was performed at the Rockefeller University Genomics Resource Center.

For the second experiment, live ants were immediately flash-frozen on dry ice. Ants were dissected into antennae, heads (without antennae), legs, and bodies (abdomen plus thorax) in 95% ethanol on dry ice, and tissues were immediately transferred to 1.5 ml tubes containing 95% ethanol, likewise on dry ice. Following dissections, ethanol was pipetted from the tubes and samples were homogenized with a Qiagen TissueLyser II in TRIzol Reagent (Sigma). The aqueous phase was then separated using the Phase Lock Gel system (5Prime) and cleaned and concentrated using the RNeasy protocol (Qiagen). cDNA libraries were constructed using Illumina TrueSeq kits and samples were run on an Illumina HiSeq 2000 platform for 100 cycles with single-end reads at the Rockefeller University Genomics Resource Center. All sequencing data is deposited in the NCBI sequence read archive (SRA accession numbers SRR1300620, SRR1477338, SRR1481486, SRR1481489, SRR1481493, SRR1481497, SRR1502786, SRR1502847, SRR1502788, SRR1502787, SRR1502848, SRR1502859, SRR1503196, and SRR1503195). Table 5 provides a summary of the samples and methods for each experiment.

**Table 5:** **Summary of experimental design for RNA-seq experiments**

Experiment	Biological replicates	Individuals pooled per replicate	Tissues sequenced	Total number of libraries	HiSeq2000 lanes	Read types	Average number of reads per library
Sex-specific antennal expression	1	25 females or 11 males	antennae (two per individual)	2	0.7	100 bp paired-end	81.5×10^6^
Tissue-specific expression in females	3	20 females	antennae (two per individual), heads, legs (six per individual), thoraces & abdomens	12	1	100 bp single-end	18×10^6^

### Expression quantification and statistical analyses

Tophat v2.0.4 [63] was used to align Illumina reads to the *C. biroi* genome (assembly v3.0, with pre-alignment to OGS 1.8.6 [41], max mismatch (-N) = 2, max intron length (-I) = 50000) and the CuffDiff algorithm of Cufflinks v2.0.2 (all parameters set to defaults) [64] was used to quantify gene expression of the predicted OBPs and CSPs (OGS 1.8.6) and test for significant tissue enrichment. Significance values reported are CuffDiff *P* values corrected for false discovery rate (the *q* value of the output file). Additional file 3 gives Cufflinks quantification for all genes in OGS 1.8.6.

### Gene sequences, re-annotation, and nomenclature

*Cerapachys biroi* sequences were obtained from our previous extensive manual annotation of *C. biroi* chemosensory genes [41]. Additionally, we used ABySS v1.3.4 [65] to build a *de novo* assembly of whole-body transcriptomic data from Oxley et al. [41] to fill-out incomplete sequences (Dryad doi pending). This led to the discovery of two additional OBPs (*CbirObp3* and *CbirObp4*) that are not present in the current genome assembly (v3.0).

Although most eusocial hymenopteran CSPs are well annotated, automatic genome annotation of OBPs is highly error prone and in many sequenced ants the OBPs are currently rather poorly annotated. We thus restricted our analyses to species with extensive manual annotation of the OBP gene family or else *de novo* assembled transcriptomic data, namely *Solenopsis invicta* [17], *Pogonomyrmex barbatus* [39], *Linepithema humile* [40], *C. biroi* [41], *A. mellifera* [25], and *Polistes Canadensis* [66]. Although high-quality CSP sequences are available for all ant species with sequenced genomes, we chose to restrict our CSP analysis to the same taxa that we used for the OBP analysis in order to make the BAli-Phy [67] analysis computationally feasible. The sequences for these taxa were obtained from Kulmuni et al. [18] Oxley et al. [41], Forêt et al. [36], and Ferreira et al. [66]. Because antennal transcript quantification data were available for the ants *Harpegnathos saltator* and *Camponotus floridanus* [4], we also manually re-annotated OBPs and CSPs in these species and included them in our phylogenetic analyses. We found several previously un-annotated OBPs in both species and one un-annotated CSP in each species. Antennal transcript quantification was not available for most of these genes (and many of the CSPs identified by Kulmuni et al. [18]) because they are not even partially represented in the official gene sets. If these genes were in single-copy orthologous groups this is noted in Figure 3. We also discovered a single-copy orthologous clade of OBPs not previously known in eusocial hymenopterans, *Obp59a* (named after the *Drosophila melanogaster* ortholog so as to allow the same name for orthologs in all species without disrupting the naming systems of Smith CR et al. [39] and Smith CD et al. [40]). Members of this clade were manually annotated for all species included in the phylogenetic analyses. Manual annotation followed Oxley et al. [41]. Additional file 4 lists all newly annotated OBPs and CSPs along with their CDS and protein sequences, genomic location, and exon structure using BED block size-block start format. For our extended insect OBP and arthropod CSP phylogenetic analyses, *Nasonia vitripennis* OBP sequences were obtained from Vieira et al. [68] and *N. vitripennis* CSP sequences from the official gene set [69], while all non-Hymenoptera sequences were obtained from Vieira and Rozas [14].

Ant CSPs have been renamed in several different studies, with the most recent and comprehensive naming system being that of Kulmuni *et al.* [18]. However, because Kulmuni *et al.*’s naming system fails to describe the orthology of ant and bee CSPs, we decided not to follow this system, but to name ant CSPs according to the previously established nomenclature for honey bee CSPs [36]. Genes in the single-copy ortholog groups were renumbered according to the *A. mellifera* ortholog when existent, and, following the identification of an additional potential single-copy ortholog group missed by Kulmuni et al. [18], all *Csp8*s were renumbered. This new group may be orthologous to *AmelCsp5*, but these proteins are so highly divergent that they do not always cluster as a clade in the maximum likelihood analyses. This gene seems to have been lost in the myrmecines and is highly pseudogenized in *L. humile*, although we did find it in *H. saltator* and *C. floridanus* and manually annotated it as mentioned above. Additional file 5 gives the translation between our CSP nomenclature and the various CSP naming systems used previously.

### Phylogenetic analyses

Although it has been hypothesized that OBPs and CSPs are homologous [14] the high sequence divergence between the two families prohibits combined phylogenetic analyses. We therefore analyzed each family separately. For each family, amino acid sequences were aligned using the MAFFT (v7.149) multiple sequence alignment algorithm using the E-INS-i strategy with the default offset value (0) for OBPs as they have multiple large gaps, and the G-INS-i strategy with an offset value of 0.123 for CSPs as they largely share global homology and are all about the same length [70]. The ProtTest server (v2.4) [71] with AICc selection criteria was used to determine the best fitting model of protein evolution for each family, in both cases the LG model [72] with gamma-distributed rate variation and a portion of invariant sites. Phylogenetic hypotheses were constructed using RAxML v8.0.22 [73], and node support was assessed using the bootstrap method with 100 bootstrap replicates. We performed these analyses both including and excluding the signal peptides using the SignalP-noTM algorithm of the SignalP v4.1 software. Topologies were nearly identical between the two treatments, differing at only a few nodes with poor support in both analyses (Additional file 6: Figure S3). Support values were also similar between the two treatments. Following Gotzek *et al.* [17], the topologies and support values shown in the main manuscript are based on the analyses including the signal peptides as these likely contain significant phylogenetic information.

Because of the highly divergent nature of OBPs and CSPs and the resulting difficulties with multiple sequence alignment (MSA), we also used the Bayesian inference software BAli-Phy (v2.1.1) [67], which co-estimates MSA with phylogeny in a Bayesian MCMC framework, thus integrating over alignment error and providing robust phylogenetic hypotheses. Two runs using default parameters and the LG + I + GAMMA model of protein evolution were conducted for each gene family. OBPs were run for 100,000 iterations, while CSPs were run for 400,000 itereations. Chain convergence was assessed by calculating potential scale reduction factors (PSRF) and average standard deviation of split frequencies (ASDSF) between runs, while effective sample size (ESS) was calculated using Tracer v1.7 [74]. For both OBPs and CSPs, PSRFs were less than 1.05, ESSs were greater than 800, and ASDSFs were less than 0.005, indicating that chains had converged [75]. All alignments and phylogenetic trees have been uploaded to the Dryad database http://doi.org/10.5061/dryad.4h56c.

### Evolutionary dynamics

Gene birth and death rates were calculated using CAFE v3.0 and JPrIME DLRS v0.2.1 [23, 24]. Because recent phylogenomic studies topologically conflict with trees used in previous Aculeata divergence dating studies [66, 76], we were forced to build an ultrametric species tree and calculate divergence times ourselves. For this, we used the single copy ant and bee genes from Oxley *et al*. [41] and found *P. canadensis* orthologs using the exonerate search algorithm [77]. We then realigned all genes with a *P. canadensis* ortholog using Muscle [78] and concatenated them into a single supergene. A maximum likelihood tree was then built using RAxML v7 [73] with the LG + Gamma model of evolution, and this tree was then made ultrametric using the r8s software by constraining the root node and using the LF method and TN algorithm [79]. All branches were then rescaled to set the best-characterized divergence (*Harpegnathos saltator* and the formicoids) to 120mya following Moreau and Bell [80]. This tree was used for all CAFE and JPrIME DLRS analyses. Our MAFFT alignments and the LG + I + Gamma model of sequence evolution were used for JPrIME DLRS. One JPrIME DLRS chain of 100,000,000 iterations was run for each group, and Tracer was used to calculate model parameter values and ESSs for each chain. All parameters had ESSs greater than 400 in all chains.

The codeml tool of paml v4.7 [26] was used to analyze selection pressures. MAFFT alignments and RAxML topologies were used in all codeml runs, with sequences and branches pruned manually when subsets were analyzed. Site models 0, 1, 2, 7, and 8 were run on both complete datasets and then each group independently. Clade model C was run on both complete datasets with each group marked as an independent clade, then compared with the M2a_rel model. For branch-site tests, all branches in a given group were marked as foreground branches with all other branches in all other groups (and between groups) left as background branches. This was done in turn for all groups. Models and model comparisons are described and discussed in [27, 81–84].

### Availability of supporting data

The data sets supporting the results of this article are available in the NCBI Sequence Read Archive under accession numbers SRR1300620, SRR1477338, SRR1481486, SRR1481489, SRR1481493, SRR1481497, SRR1502786, SRR1502847, SRR1502788, SRR1502787, SRR1502848, SRR1502859, SRR1503196, and SRR1503195 and the Dryad database http://doi.org/10.5061/dryad.4h56c.

## Electronic supplementary material

Additional file 1: Figure S1: Maximum likelihood phylogeny of insect odorant binding proteins. Constructed using RAxML with the GAMMA + I + LG evolutionary model from protein sequences aligned using the E-INS-I algorithm of MAFFT. Branches are colored by taxonomic order: Green: Hemiptera; brown: Psocodea; blue: Hymenoptera; orange: Coleoptera; pink: Lepidoptera; red: Diptera. (PDF 472 KB)

Additional file 2: Figure S2: Maximum likelihood phylogeny of arthropod chemosensory proteins. Constructed using RAxML with the GAMMA + I + LG evolutionary model from protein sequences aligned using the G-INS-I algorithm of MAFFT. Branches are colored by taxonomic order: Yellow: Ixodida (Arachnida); turquoise: Cladocera (Branchiopoda); green: Hemiptera; brown: Psocodea; blue: Hymenoptera; orange: Coleoptera; pink: Lepidoptera; red: Diptera. (PDF 10 KB)

Additional file 3: Cufflinks quantification of expression for all RNA-seq libraries for all genes in OGS 1.8.6. (CSV 7 MB)

Additional file 4: All newly-annotated odorant binding protein and chemosensory protein genes. CDS: coding nucleotide sequence; Prot: amino acid sequence; Loc: genomic/transcriptomics locus; strand: coding strand; BEDsize: length of each exon (# nucleotides); BEDstart: locus position before first nucleotide of each exon. (CSV 33 KB)

Additional file 5: Names of aculeate chemosensory protein genes according to various naming systems, along with amino acid sequences. (CSV 21 KB)

Additional file 6: Figure S3: Trees and bootstrap support values from RAxML analyses with signal peptides included as well as excluded, as well as Bali-Phy analyses consensus tree with posterior probabilities. (PDF 510 KB)
