# Supplementary figures and images for: Comparative genomics and transcriptomics in ants provide new insights into the evolution and function of odorant binding and chemosensory proteins
Source: BMC Genomics. 2014 Aug 26;15(1):718. doi: 10.1186/1471-2164-15-718 (PMC4161878; doi:10.1186/1471-2164-15-718)

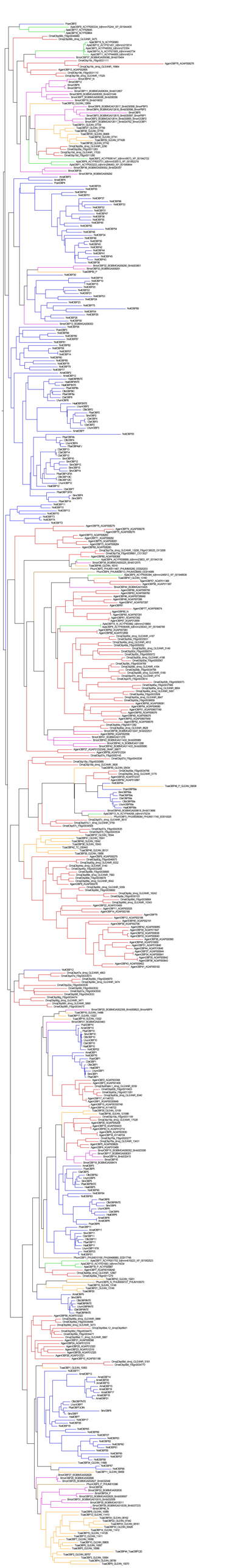

Supplement: Supplementary file 1 — Additional file 1: Figure S1: Maximum likelihood phylogeny of insect odorant binding proteins. Constructed using RAxML with the GAMMA + I + LG evolutionary model from protein sequences aligned using the E-INS-I algorithm of MAFFT. Branches are colored by taxonomic order: Green: Hemiptera; brown: Psocodea; blue: Hymenoptera; orange: Coleoptera; pink: Lepidoptera; red: Diptera. (PDF 472 KB) [file 12864_2014_6421_MOESM1_ESM.pdf]

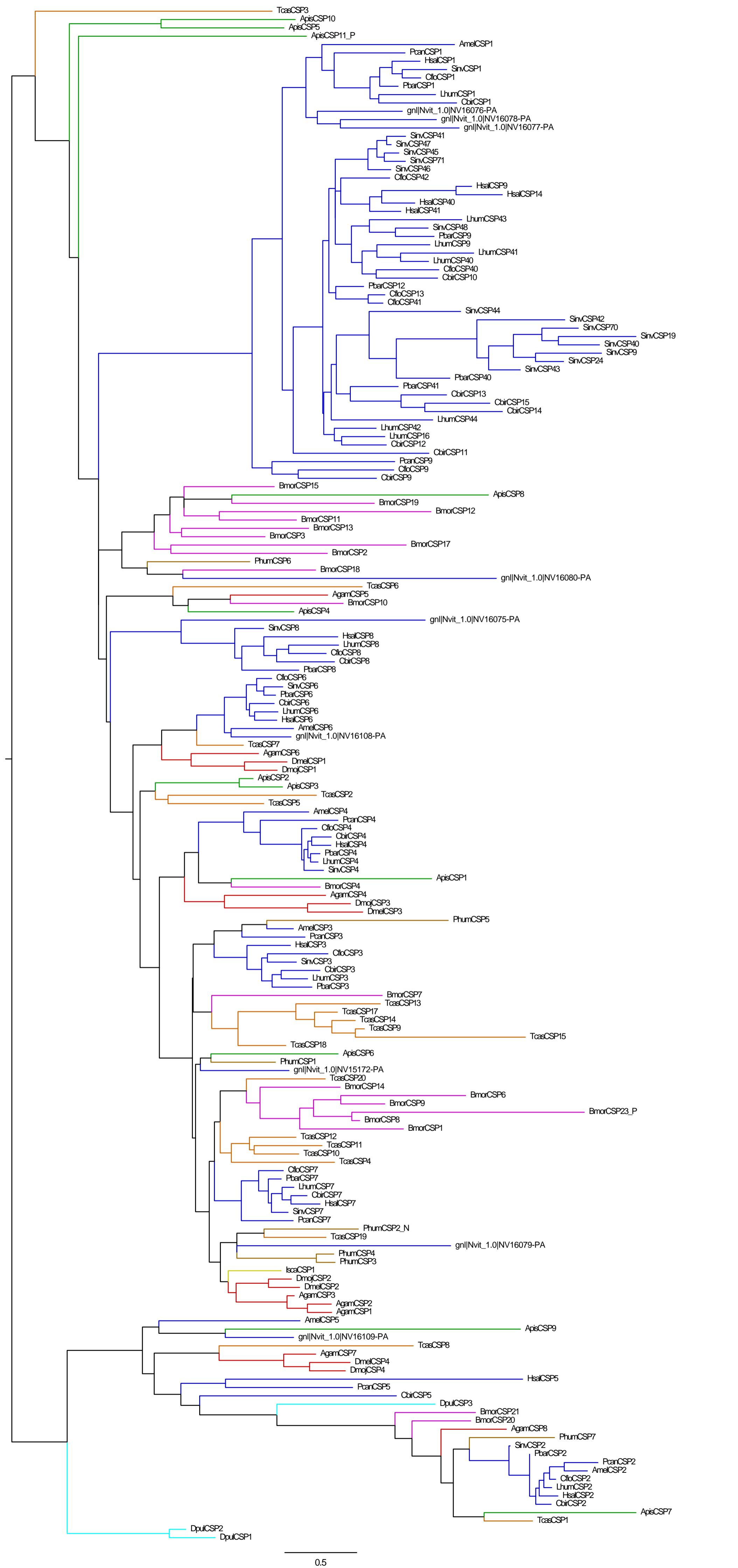

Supplement: Supplementary file 2 — Additional file 2: Figure S2: Maximum likelihood phylogeny of arthropod chemosensory proteins. Constructed using RAxML with the GAMMA + I + LG evolutionary model from protein sequences aligned using the G-INS-I algorithm of MAFFT. Branches are colored by taxonomic order: Yellow: Ixodida (Arachnida); turquoise: Cladocera (Branchiopoda); green: Hemiptera; brown: Psocodea; blue: Hymenoptera; orange: Coleoptera; pink: Lepidoptera; red: Diptera. (PDF 10 KB) [file 12864_2014_6421_MOESM2_ESM.pdf]
