# Supplementary material for: Comparative genomics and transcriptomics in ants provide new insights into the evolution and function of odorant binding and chemosensory proteins
Source: BMC Genomics. 2014 Aug 26;15(1):718. doi: 10.1186/1471-2164-15-718 (PMC4161878; doi:10.1186/1471-2164-15-718)
Supplement: Supplementary file 6 — Additional file 6: Figure S3: Trees and bootstrap support values from RAxML analyses with signal peptides included as well as excluded, as well as Bali-Phy analyses consensus tree with posterior probabilities. (PDF 510 KB) [file 12864_2014_6421_MOESM6_ESM.pdf]

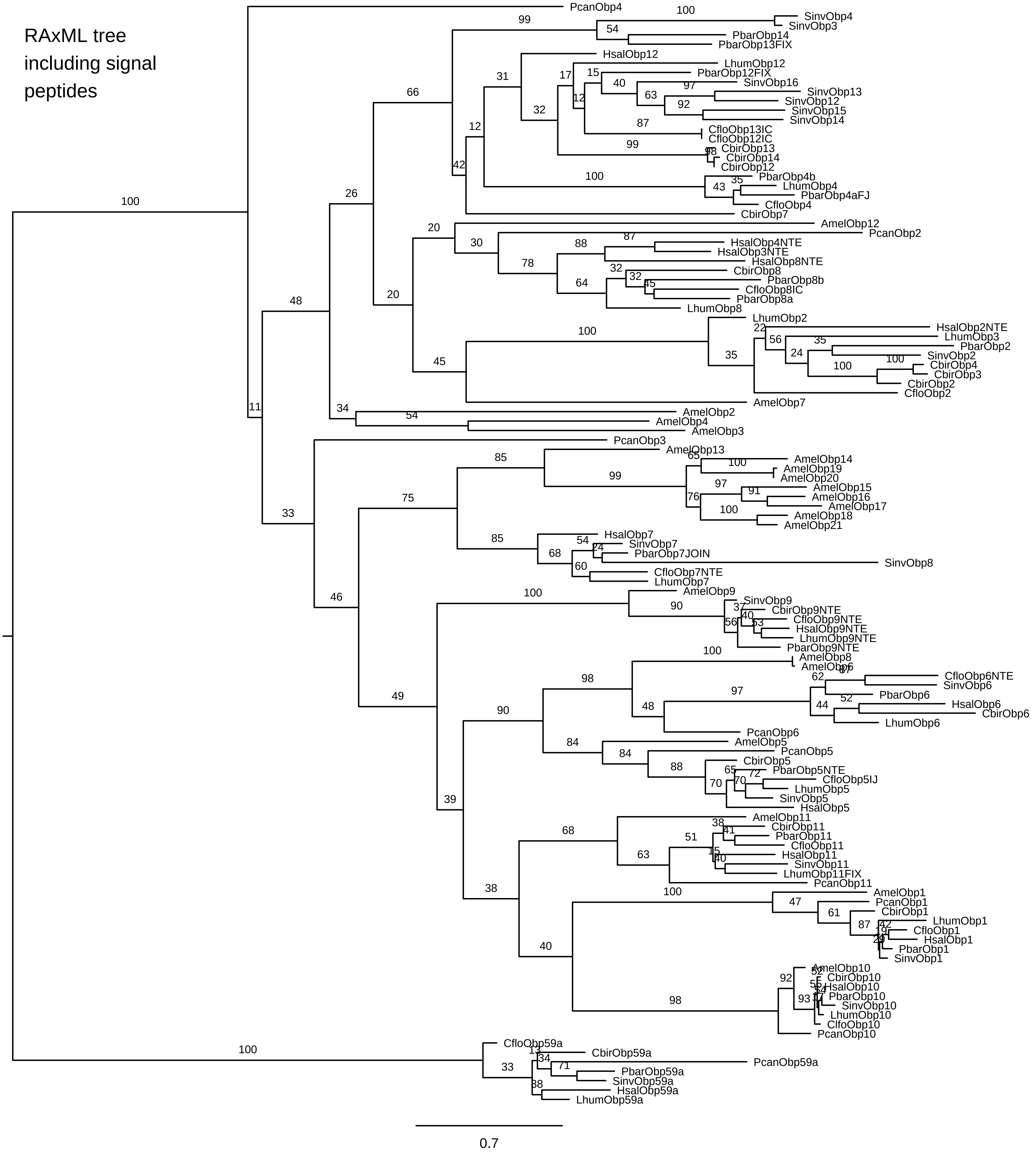
RAxML tree
including signal
peptides
PcanObp4
SinvObp4
SinvObp3
PbarObp14
PbarObp13FIX
HsalObp12
LhumObp12
PbarObp12FIX
SinvObp16
SinvObp13
SinvObp12
SinvObp15
SinvObp14
CfloObp13IC
CfloObp12IC
CbirObp13
CbirObp14
CbirObp12
PbarObp4b
LhumObp4
PbarObp4aFJ
CfloObp4
CbirObp7
AmelObp12
PcanObp2
HsalObp4NTE
HsalObp3NTE
HsalObp8NTE
CbirObp8
PbarObp8b
CfloObp8IC
PbarObp8a
LhumObp8
LhumObp2
HsalObp2NTE
LhumObp3
PbarObp2
SinvObp2
CbirObp4
CbirObp3
CbirObp2
CfloObp2
AmelObp7
AmelObp2
AmelObp4
AmelObp3
PcanObp3
AmelObp13
AmelObp14
AmelObp19
AmelObp20
AmelObp15
AmelObp16
AmelObp17
AmelObp18
AmelObp21
HsalObp7
SinvObp7
PbarObp7JOIN
SinvObp8
CfloObp7NTE
LhumObp7
AmelObp9
SinvObp9
CbirObp9NTE
CfloObp9NTE
HsalObp9NTE
LhumObp9NTE
PbarObp9NTE
AmelObp8
AmelObp6
CfloObp6NTE
SinvObp6
PbarObp6
HsalObp6
CbirObp6
LhumObp6
PcanObp6
AmelObp5
PcanObp5
CbirObp5
PbarObp5NTE
CfloObp5IJ
LhumObp5
SinvObp5
HsalObp5
AmelObp11
CbirObp11
PbarObp11
CfloObp11
HsalObp11
SinvObp11
LhumObp11FIX
PcanObp11
AmelObp1
PcanObp1
CbirObp1
LhumObp1
CfloObp1
HsalObp1
PbarObp1
SinvObp1
AmelObp10
CbirObp10
HsalObp10
PbarObp10
SinvObp10
LhumObp10
ClfoObp10
PcanObp10
CfloObp59a
CbirObp59a
PcanObp59a
PbarObp59a
SinvObp59a
HsalObp59a
LhumObp59a
0.7

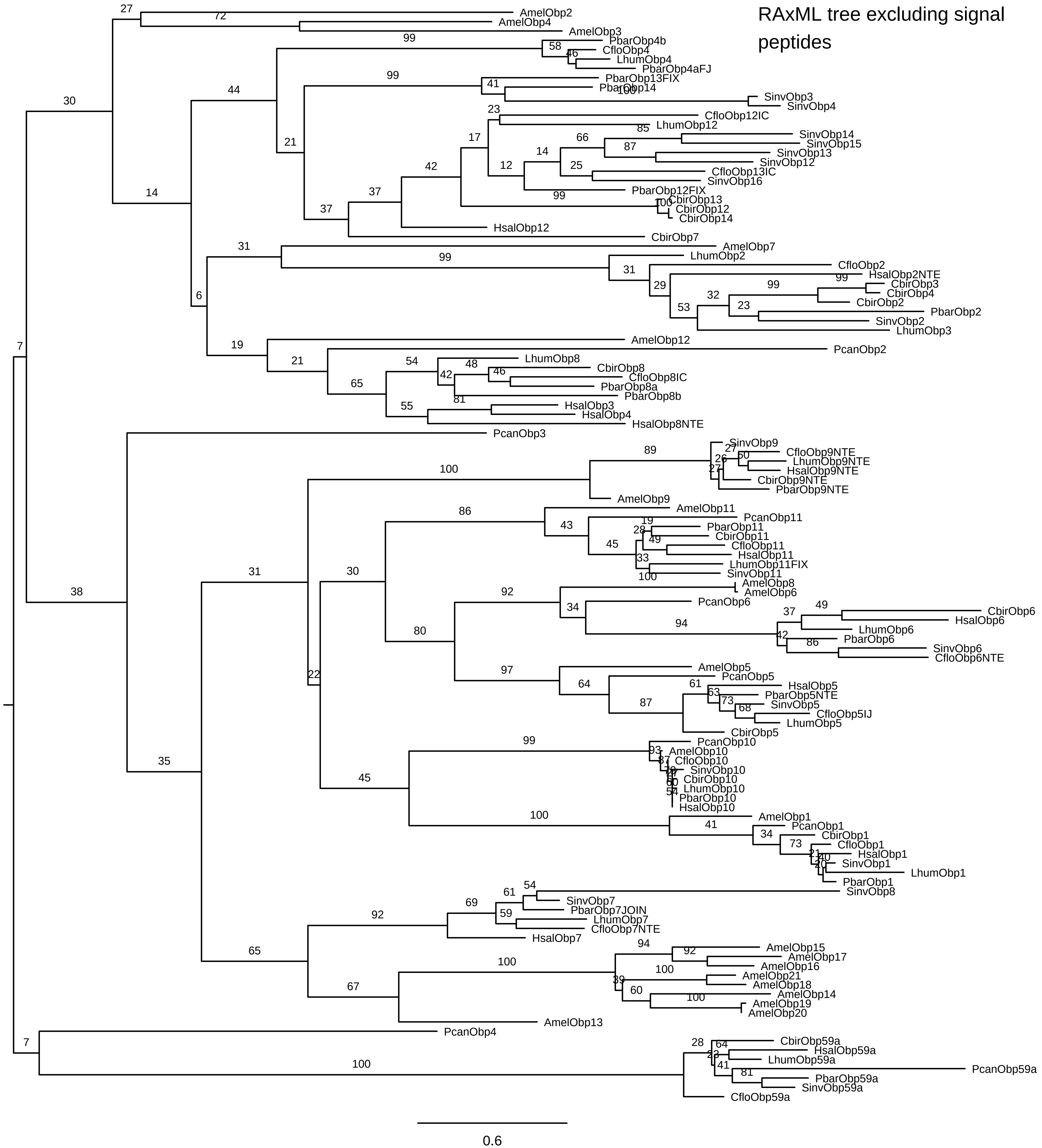
RAxML tree excluding signal peptides
AmelObp2
AmelObp4
AmelObp3
PbarObp4b
CfloObp4
LhumObp4
PbarObp4aFJ
PbarObp13FIX
PbarObp14
SinvObp3
SinvObp4
CfloObp12IC
LhumObp12
SinvObp14
SinvObp15
SinvObp13
SinvObp12
CfloObp13IC
SinvObp16
PbarObp12FIX
CbirObp13
CbirObp12
CbirObp14
HsalObp12
CbirObp7
AmelObp7
LhumObp2
CfloObp2
HsalObp2NTE
CbirObp3
CbirObp4
CbirObp2
PbarObp2
SinvObp2
LhumObp3
AmelObp12
PcanObp2
LhumObp8
CbirObp8
CfloObp8IC
PbarObp8a
PbarObp8b
HsalObp3
HsalObp4
HsalObp8NTE
PcanObp3
SinvObp9
CfloObp9NTE
LhumObp9NTE
HsalObp9NTE
CbirObp9NTE
PbarObp9NTE
AmelObp9
AmelObp11
PcanObp11
PbarObp11
CbirObp11
CfloObp11
HsalObp11
LhumObp11FIX
SinvObp11
AmelObp8
AmelObp6
PcanObp6
CbirObp6
HsalObp6
LhumObp6
PbarObp6
SinvObp6
CfloObp6NTE
AmelObp5
PcanObp5
HsalObp5
PbarObp5NTE
SinvObp5
CfloObp5IJ
LhumObp5
CbirObp5
PcanObp10
AmelObp10
CfloObp10
SinvObp10
CbirObp10
LhumObp10
PbarObp10
HsalObp10
AmelObp1
PcanObp1
CbirObp1
CfloObp1
HsalObp1
SinvObp1
LhumObp1
PbarObp1
SinvObp8
SinvObp7
PbarObp7JOIN
LhumObp7
CfloObp7NTE
HsalObp7
AmelObp15
AmelObp17
AmelObp16
AmelObp21
AmelObp18
AmelObp14
AmelObp19
AmelObp20
AmelObp13
PcanObp4
CbirObp59a
HsalObp59a
LhumObp59a
PcanObp59a
PbarObp59a
SinvObp59a
CfloObp59a
0.6

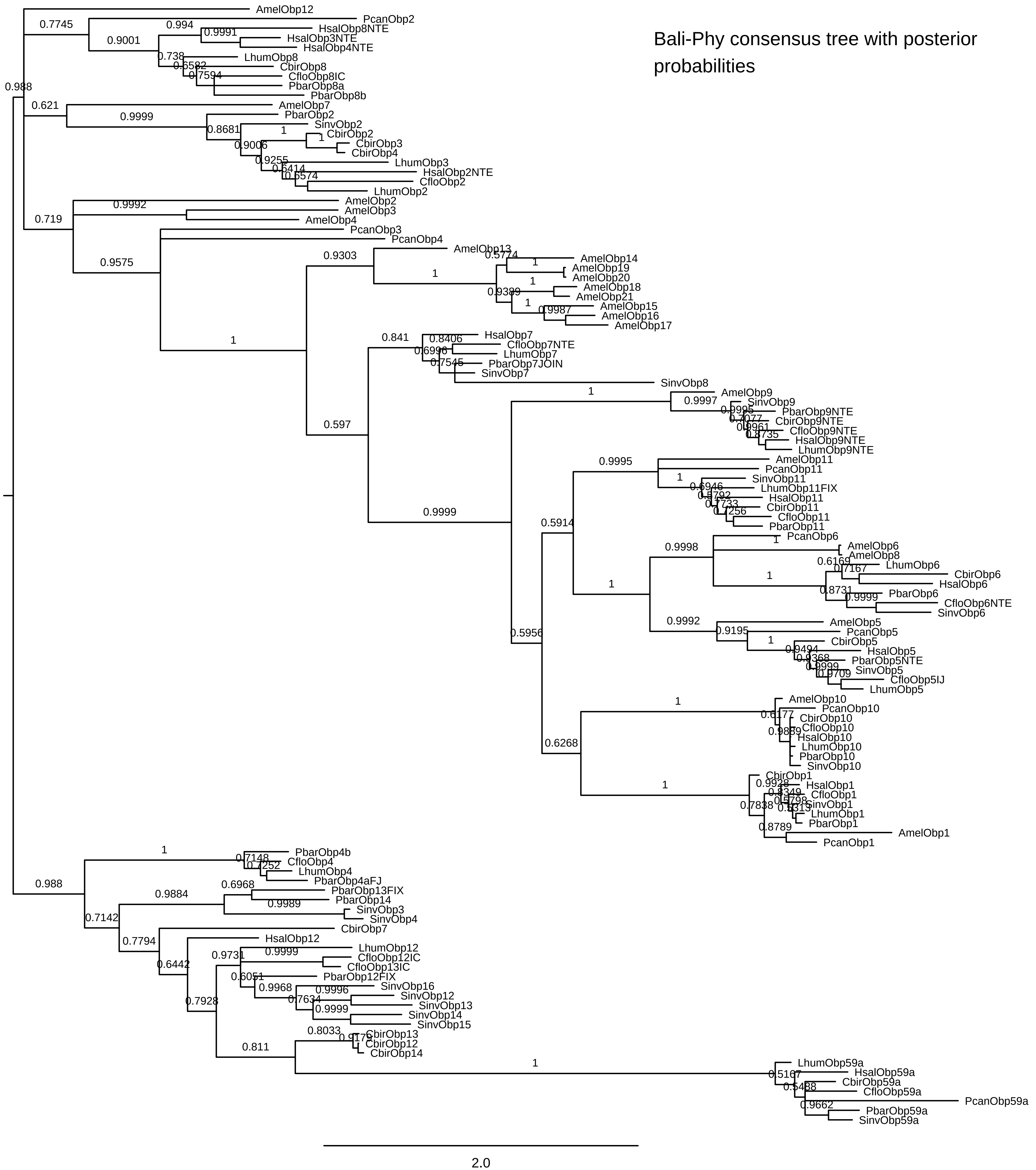
Bali-Phy consensus tree with posterior probabilities
AmelObp12
PcanObp2
HsalObp8NTE
HsalObp3NTE
HsalObp4NTE
LhumObp8
CbirObp8
CfloObp8IC
PbarObp8a
PbarObp8b
AmelObp7
PbarObp2
SinvObp2
CbirObp2
CbirObp3
CbirObp4
LhumObp3
HsalObp2NTE
CfloObp2
LhumObp2
AmelObp2
AmelObp3
AmelObp4
PcanObp3
PcanObp4
AmelObp13
AmelObp14
AmelObp19
AmelObp20
AmelObp18
AmelObp21
AmelObp15
AmelObp16
AmelObp17
HsalObp7
CfloObp7NTE
LhumObp7
PbarObp7JOIN
SinvObp7
SinvObp8
AmelObp9
SinvObp9
PbarObp9NTE
CbirObp9NTE
CfloObp9NTE
HsalObp9NTE
LhumObp9NTE
AmelObp11
PcanObp11
SinvObp11
LhumObp11FIX
HsalObp11
CbirObp11
CfloObp11
PbarObp11
PcanObp6
AmelObp6
AmelObp8
LhumObp6
CbirObp6
HsalObp6
PbarObp6
CfloObp6NTE
SinvObp6
AmelObp5
PcanObp5
CbirObp5
HsalObp5
PbarObp5NTE
SinvObp5
CfloObp5IJ
LhumObp5
AmelObp10
PcanObp10
CbirObp10
CfloObp10
HsalObp10
LhumObp10
PbarObp10
SinvObp10
CbirObp1
HsalObp1
CfloObp1
SinvObp1
LhumObp1
PbarObp1
AmelObp1
PcanObp1
PbarObp4b
CfloObp4
LhumObp4
PbarObp4aFJ
PbarObp13FIX
PbarObp14
SinvObp3
SinvObp4
CbirObp7
HsalObp12
LhumObp12
CfloObp12IC
CfloObp13IC
PbarObp12FIX
SinvObp16
SinvObp12
SinvObp13
SinvObp14
SinvObp15
CbirObp13
CbirObp12
CbirObp14
LhumObp59a
HsalObp59a
CbirObp59a
CfloObp59a
PcanObp59a
PbarObp59a
SinvObp59a
2.0

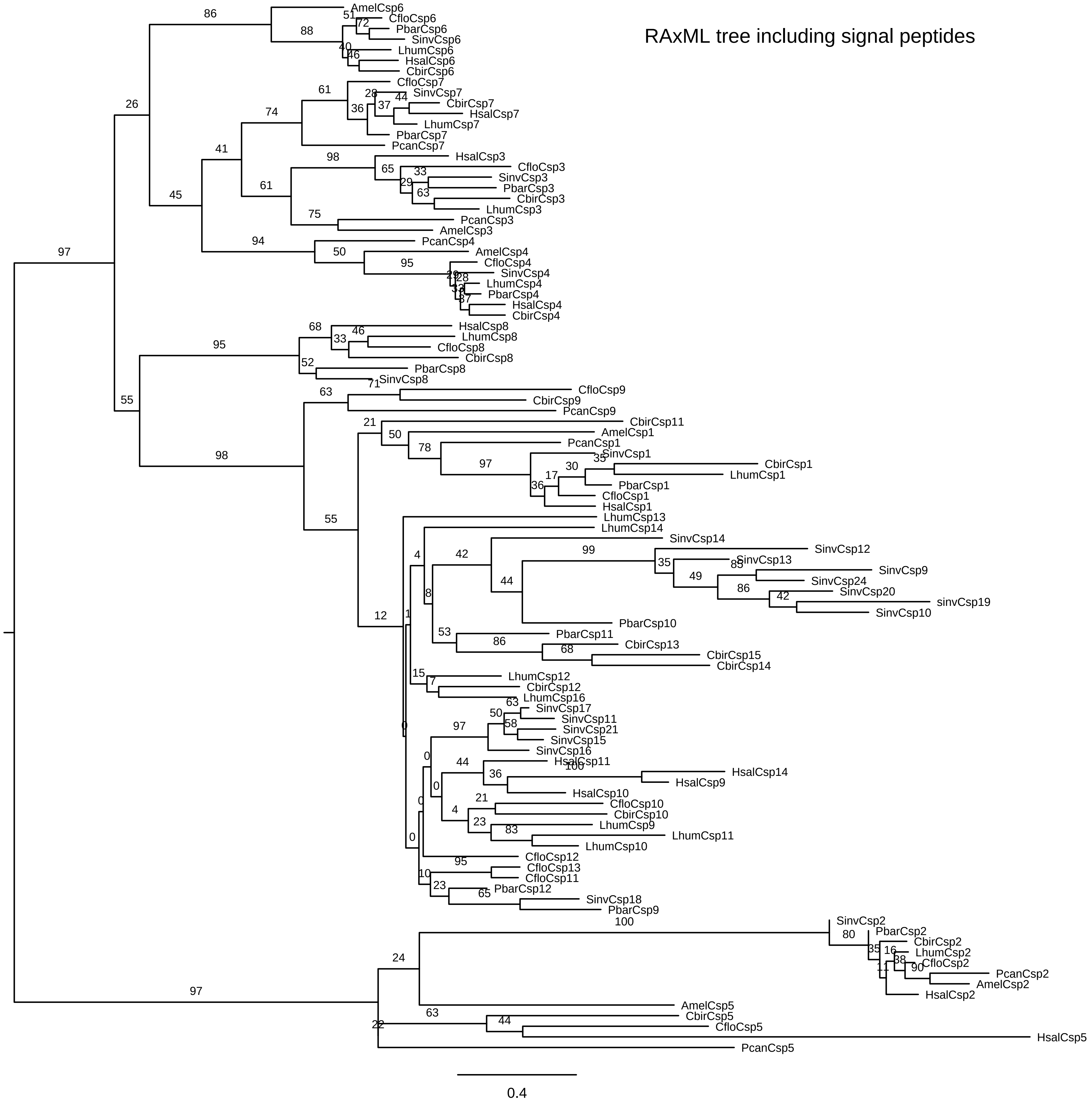
RAxML tree including signal peptides
AmelCsp6
CfloCsp6
PbarCsp6
SinvCsp6
LhumCsp6
HsalCsp6
CbirCsp6
CfloCsp7
SinvCsp7
CbirCsp7
HsalCsp7
LhumCsp7
PbarCsp7
PcanCsp7
HsalCsp3
CfloCsp3
SinvCsp3
PbarCsp3
CbirCsp3
LhumCsp3
PcanCsp3
AmelCsp3
PcanCsp4
AmelCsp4
CfloCsp4
SinvCsp4
LhumCsp4
PbarCsp4
HsalCsp4
CbirCsp4
HsalCsp8
LhumCsp8
CfloCsp8
CbirCsp8
PbarCsp8
SinvCsp8
CfloCsp9
CbirCsp9
PcanCsp9
CbirCsp11
AmelCsp1
PcanCsp1
SinvCsp1
CbirCsp1
LhumCsp1
PbarCsp1
CfloCsp1
HsalCsp1
LhumCsp13
LhumCsp14
SinvCsp14
SinvCsp12
SinvCsp13
SinvCsp9
SinvCsp24
SinvCsp20
sinvCsp19
SinvCsp10
PbarCsp10
PbarCsp11
CbirCsp13
CbirCsp15
CbirCsp14
LhumCsp12
CbirCsp12
LhumCsp16
SinvCsp17
SinvCsp11
SinvCsp21
SinvCsp15
SinvCsp16
HsalCsp11
HsalCsp14
HsalCsp9
HsalCsp10
CfloCsp10
CbirCsp10
LhumCsp9
LhumCsp11
LhumCsp10
CfloCsp12
CfloCsp13
CfloCsp11
PbarCsp12
SinvCsp18
PbarCsp9
SinvCsp2
PbarCsp2
CbirCsp2
LhumCsp2
CfloCsp2
PcanCsp2
AmelCsp2
HsalCsp2
AmelCsp5
CbirCsp5
CfloCsp5
HsalCsp5
PcanCsp5
0.4

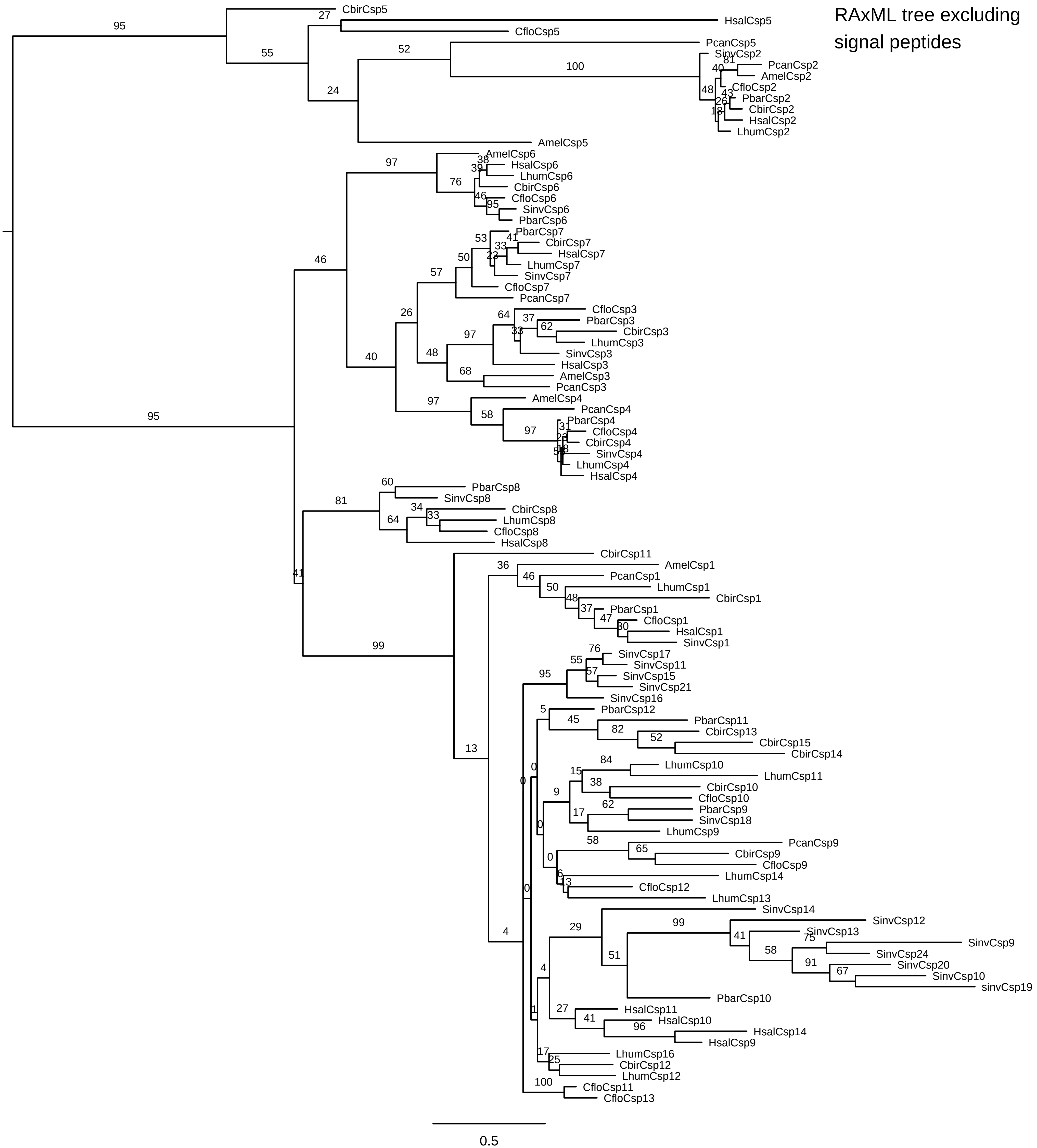
RAxML tree excluding signal peptides
CbirCsp5
HsalCsp5
CfloCsp5
PcanCsp5
SinvCsp2
PcanCsp2
AmelCsp2
CfloCsp2
PbarCsp2
CbirCsp2
HsalCsp2
LhumCsp2
AmelCsp5
AmelCsp6
HsalCsp6
LhumCsp6
CbirCsp6
CfloCsp6
SinvCsp6
PbarCsp6
PbarCsp7
CbirCsp7
HsalCsp7
LhumCsp7
SinvCsp7
CfloCsp7
PcanCsp7
CfloCsp3
PbarCsp3
CbirCsp3
LhumCsp3
SinvCsp3
HsalCsp3
AmelCsp3
PcanCsp3
AmelCsp4
PcanCsp4
PbarCsp4
CfloCsp4
CbirCsp4
SinvCsp4
LhumCsp4
HsalCsp4
PbarCsp8
SinvCsp8
CbirCsp8
LhumCsp8
CfloCsp8
HsalCsp8
CbirCsp11
AmelCsp1
PcanCsp1
LhumCsp1
CbirCsp1
PbarCsp1
CfloCsp1
HsalCsp1
SinvCsp1
SinvCsp17
SinvCsp11
SinvCsp15
SinvCsp21
SinvCsp16
PbarCsp12
PbarCsp11
CbirCsp13
CbirCsp15
CbirCsp14
LhumCsp10
LhumCsp11
CbirCsp10
CfloCsp10
PbarCsp9
SinvCsp18
LhumCsp9
PcanCsp9
CbirCsp9
CfloCsp9
LhumCsp14
CfloCsp12
LhumCsp13
SinvCsp14
SinvCsp12
SinvCsp13
SinvCsp9
SinvCsp24
SinvCsp20
SinvCsp10
sinvCsp19
PbarCsp10
HsalCsp11
HsalCsp10
HsalCsp14
HsalCsp9
LhumCsp16
CbirCsp12
LhumCsp12
CfloCsp11
CfloCsp13
0.5

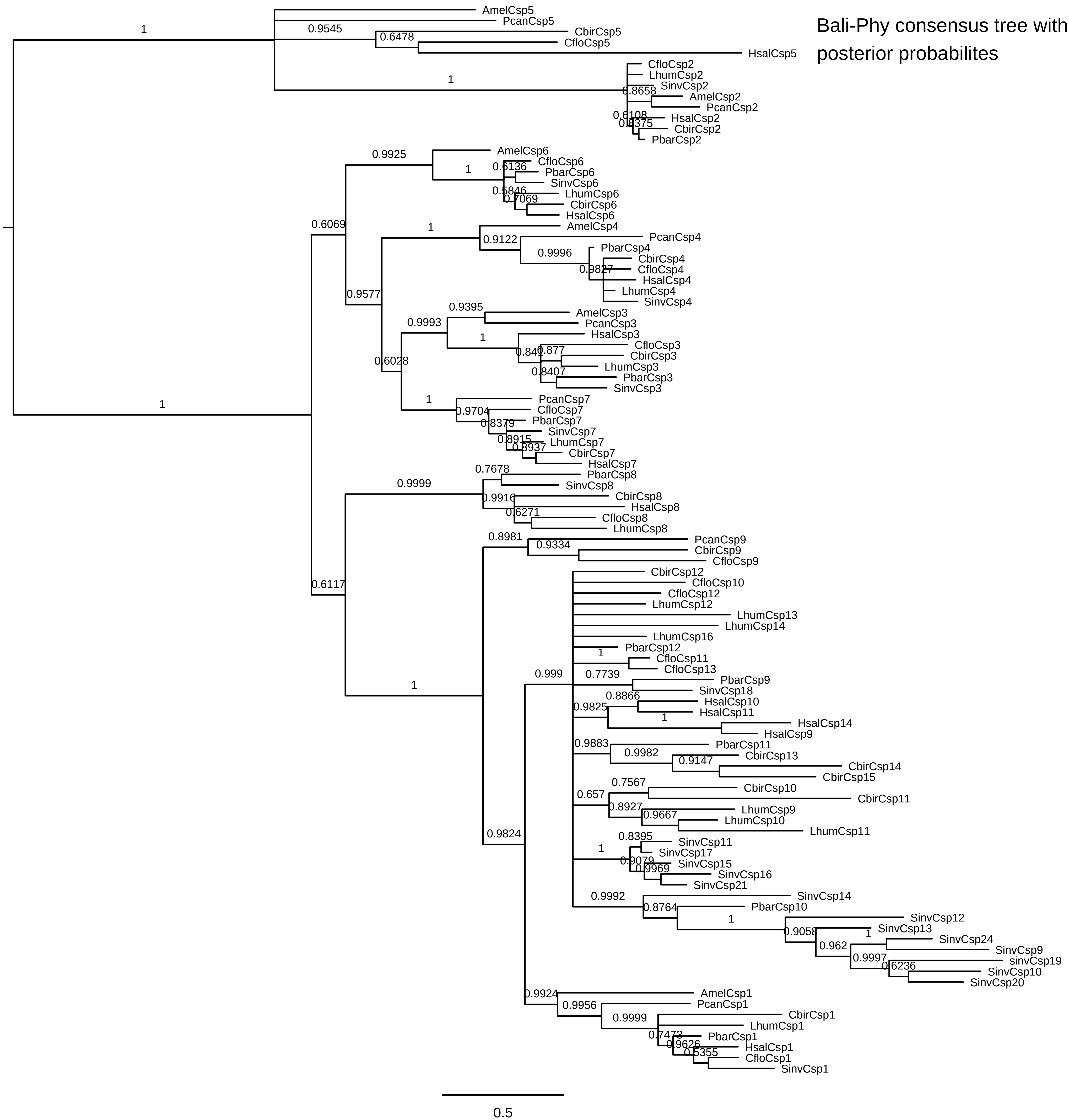

Bali-Phy consensus tree with posterior probabilites
